# Machine-learning model predicting postoperative delirium in older patients using intraoperative frontal electroencephalographic signatures

**DOI:** 10.3389/fnagi.2022.911088

**Published:** 2022-10-14

**Authors:** Vera Röhr, Benjamin Blankertz, Finn M. Radtke, Claudia Spies, Susanne Koch

**Affiliations:** ^1^Neurotechnology Group, Technische Universität Berlin, Berlin, Germany; ^2^Department of Anaesthesia, Hospital of Nykobing, University of Southern Denmark, Odense, Denmark; ^3^Department of Anaesthesiology and Operative Intensive Care Medicine, Charité—Universitätsmedizin Berlin, Berlin, Germany

**Keywords:** electroencephalography, artificial intelligence, prediction model, machine learning, aging, postoperative delirium, surgery

## Abstract

**Objective:**

In older patients receiving general anesthesia, postoperative delirium (POD) is the most frequent form of cerebral dysfunction. Early identification of patients at higher risk to develop POD could provide the opportunity to adapt intraoperative and postoperative therapy. We, therefore, propose a machine learning approach to predict the risk of POD in elderly patients, using routine intraoperative electroencephalography (EEG) and clinical data that are readily available in the operating room.

**Methods:**

We conducted a retrospective analysis of the data of a single-center study at the Charité-Universitätsmedizin Berlin, Department of Anesthesiology [ISRCTN 36437985], including 1,277 patients, older than 60 years with planned surgery and general anesthesia. To deal with the class imbalance, we used balanced ensemble methods, specifically Bagging and Random Forests and as a performance measure, the area under the ROC curve (AUC-ROC). We trained our models including basic clinical parameters and intraoperative EEG features in particular classical spectral and burst suppression signatures as well as multi-band covariance matrices, which were classified, taking advantage of the geometry of a Riemannian manifold. The models were validated with 10 repeats of a 10-fold cross-validation.

**Results:**

Including EEG data in the classification resulted in a robust and reliable risk evaluation for POD. The clinical parameters alone achieved an AUC-ROC score of 0.75. Including EEG signatures improved the classification when the patients were grouped by anesthetic agents and evaluated separately for each group. The spectral features alone showed an AUC-ROC score of 0.66; the covariance features showed an AUC-ROC score of 0.68. The AUC-ROC scores of EEG features relative to patient data differed by anesthetic group. The best performance was reached, combining both the EEG features and the clinical parameters. Overall, the AUC-ROC score was 0.77, for patients receiving Propofol it was 0.78, for those receiving Sevoflurane it was 0.8 and for those receiving Desflurane 0.73. Applying the trained prediction model to an independent data set of a different clinical study confirmed these results for the combined classification, while the classifier on clinical parameters alone did not generalize.

**Conclusion:**

A machine learning approach combining intraoperative frontal EEG signatures with clinical parameters could be an easily applicable tool to early identify patients at risk to develop POD.

## 1. Introduction

Postoperative delirium (POD) is a common complication and the most frequent cerebral dysfunction after surgery requiring general anesthesia among elderly patients. It manifests as a disturbance of consciousness, attention, perception, memory, and cognition as well as a disruption of the sleep-wake rhythm and can lead to adverse long-term complications such as increased mortality, prolonged hospital stays, and persisting cognitive impairments (Aldecoa et al., 2017). Even though it is such a common condition with severe complications, it is often overlooked. This is mainly caused by the predominant hypoactive motor aspect of POD in older patients, hence not being noticed by physicians and nurses on a busy peripheral ward. Early identification of patients at high risk for POD could significantly improve the clinical routine in postoperative care. Based on a reliable prediction tool, patients with higher risks could be the focus of monitoring and prevention, decreasing the daily workload to those patients of concern.

Predisposing risk factors for POD have been identified as frailty, aging, lower cognitive abilities, and preexisting co-morbidities (Aldecoa et al., 2017; Culley et al., 2017). In addition to these classical risk factors, several machine learning approaches have been developed for predicting the risk of POD with the goal of early diagnosis and prevention during or directly after surgery. These approaches rely on different databases, such as electronic health record data (Wang et al., 2020; Bishara et al., 2022) or MRI data (Kyeong et al., 2018) and often focus on one type of surgery (Kyeong et al., 2018; Wang et al., 2020). EEG data has been used as well (van Sleuwen et al., 2022; Tesh et al., 2022), however, not to predict POD specifically, but to predict clinical outcomes and the severity of delirium in general for diagnosis and treatment once patients might have developed delirium on the ward.

There have not been machine learning models using routine intraoperative EEG monitoring data, even though EEG has shown characteristic signatures during surgery connected to the classical risk factors. On the one hand, preexisting cognitive dysfunction in older patients is associated with reduced intraoperative alpha-band power (Gutierrez et al., 2019; Koch et al., 2019). On the other hand, patients at higher age more readily present intraoperative burst suppression activity and show reduced intraoperative alpha-band power (Purdon et al., 2015). Both of these are associated with a higher risk to develop POD (Soehle et al., 2015; Fritz et al., 2016; Koch et al., 2021).

In the present study, we use the raw EEG files from the BIS Neuromonitor (BISTMMedtronic). We complement the EEG data with clinical patient data that is routinely available in the operating room. Apart from the medication directly connected to the surgery, we only take the American Society of Anesthesiology (ASA) score, the age of the patient, and the duration of the operation into account. On this basis, we aim to develop a robust machine learning model to predict POD, showing that incorporating EEG signatures improves the risk evaluation.

## 2. Methods

To predict the risk of developing POD, we retrospectively analyzed the intraoperative raw EEG files from the single-center study SuDoCo at the Charité-Universitätsmedizin Berlin, Department of Anesthesiology [ISRCTN 36437985] (Radtke et al., 2013). The study included 1,277 patients and POD was diagnosed based on the Diagnostic and Statistical Manual of Mental Disorders (DSM IV) assessments twice daily starting in the recovery room until the evening of postoperative day 7. The patients were labeled as POD patients if POD was diagnosed in at least one of the assessments. To ensure patients had no delirium before the surgery, they underwent Mini-Mental State Examination, and patients who had scores <24 were excluded from the overall study. The anesthetic procedure was not controlled by the study regime, and anesthesiologists conducted general anesthesia according to the standard operating procedures of the Charité-Universitätsmedizin Berlin.

Intraoperative frontal EEG channels (Fp1, Fp2, F7, and F8) used by the BIS monitor were recorded (Radtke et al., 2013). Since we used only the raw EEG recordings, there were no event markers in the EEG. Our analysis does not refer to any processed EEG parameters given in the BIS monitor by any inbuilt algorithms. The recordings vary in length, 254 of the recordings were partial recordings of the operation. We excluded any patient with missing EEG recordings or that had <20 min of intraoperative EEG data left after preprocessing, which will be discussed in the next subsection. Additionally, we excluded patients with missing clinical data.

During preprocessing, we extracted the burst suppression features for each patient from the EEG data (see Section 2.1), as well as two time-dependent features: the spectral features and the covariance features (explained in detail in Section 2.2.1). The latter two represent a sequence of 2 min time frames of the EEG data. The spectral features contain the frequency spectrum for each time frame, while the covariance features contain multi-band covariance matrices for each time frame. The two time-dependent features are classified separately. For some steps in the preprocessing (Section 2.1) and the classification of the covariance features, we use the Riemannian framework (Barachant et al., 2013; Congedo et al., 2017; Congedo, 2018; Barthélemy et al., 2019). All the data analysis is performed in the programming language *Julia*, importing the python packages *imbalanced-learn* (Lemaître et al., 2017) and *scikit-learn* (Pedregosa et al., 2011).

**Figure 1:**
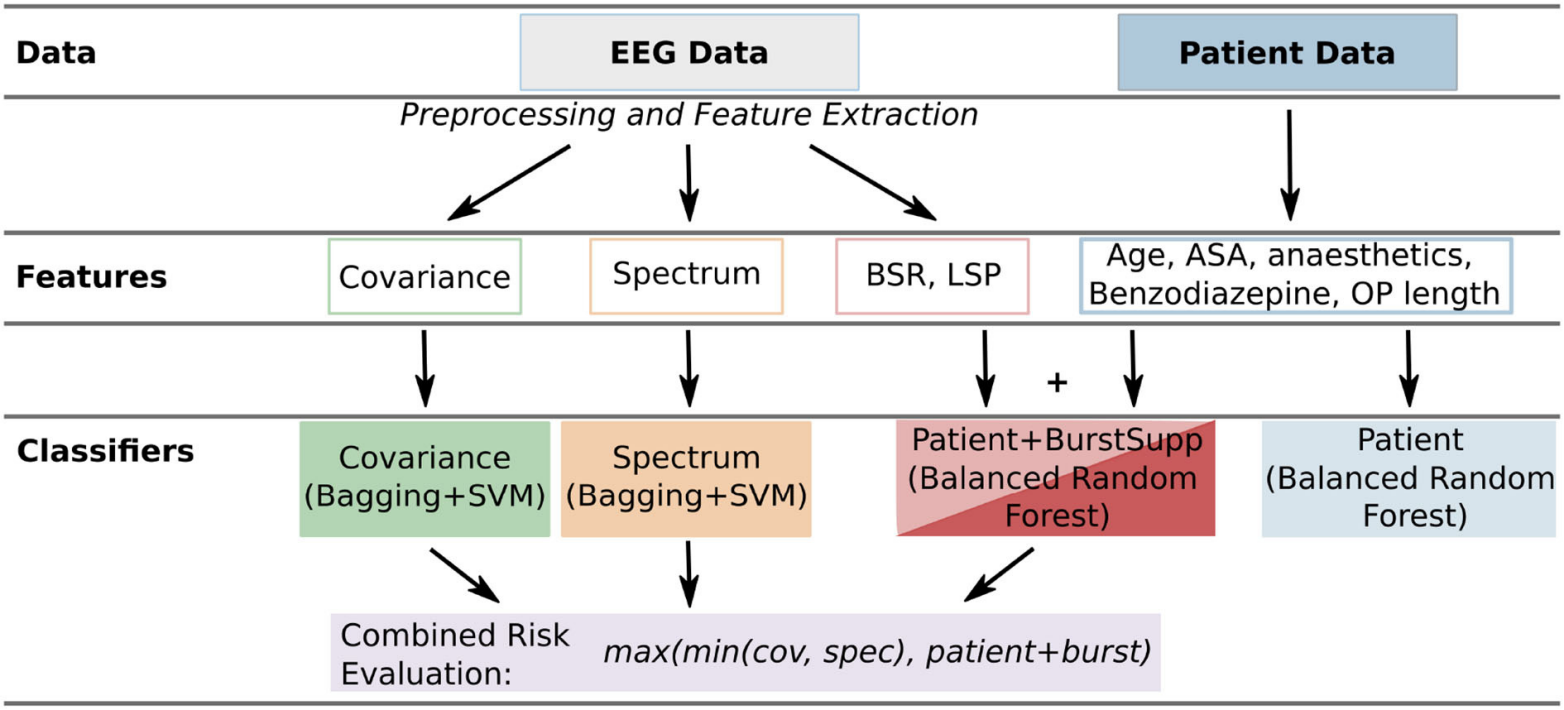
Overview of the used features and classifiers. For each patient, there are two general types of data: the EEG data and the patient data. From the EEG data, three types of features [time-dependent: covariance and spectrum, numerical: burst suppression ratio (BSR), and longest suppression phase (LSP)] are extracted (Section 2.1). From the patient data, the data easily available in the operating room is used. These features are classified by different classifiers (covariance, spectrum, patient+burstsupp, combined risk evaluation, and patient data classifier; Section 2.2) and then evaluated and compared (Section 3).

The burst suppression features were added to the clinical patient data and then classified. The following features of the clinical patient data were used: the administered anesthetic agents (categorical), whether a patient received benzodiazepine (binary), the age (numeric), the length of the operation (numeric), and the ASA score (numeric). The three classifiers corresponding to the categories of features were then combined into a final risk evaluation (Figure 1).

### 2.1. EEG data preprocessing and feature extraction

We focus on two main types of artifacts: high amplitude artifacts and frequency artifacts. For the first category, we use an amplitude filter, excluding high amplitude artifacts. Since we have different subjects and settings under which the data was recorded, the amplitudes scale differently in different recordings. Hence, we do not define a fixed threshold to exclude the amplitude artifacts but use the 99% quantile of the amplitudes for each patient. The data is re-referenced by the mean (common average reference). Afterward, we apply a bandpass filter (0.3–50 Hz) for the frequency artifacts.

After applying the two filters, the data is automatically segmented (see Supplementary Section S1.1). The segmentation takes the amplitude artifacts and potential edge artifacts introduced by the band-pass filters into account, leaving us with a series of separated time segments between artifacts. Any segments smaller than 1s are removed from the series to ensure an accurate estimation of the spectrum and covariance matrix for each segment.

After z-scoring the data, the estimation of the spectral density or frequency spectrum is done using the Welch method (Welch, 1967; Congedo, 2018) for each segment. This is the basis for the spectral features. Furthermore, we extract additional intraoperative signatures (Purdon et al., 2013), namely the burst suppression ratio (BSR) and the longest suppression phase (LSP), and calculate burst suppression probability for each time point, estimating a burst suppression timeline (Supplementary Section S1.3). Burst suppression is an EEG pattern, where periods of almost no EEG activity alternate with periods of high activity. To calculate the BSR, we determined the suppression time in the EEG—with very little to no activity—and we divided this by the duration of the time interval recorded, which in our case was the length of the recorded EEG file reduced by the removed artifacts.

For the covariance features, we use a multi-band signal. To calculate the multi-band signal, we filter the EEG data to the delta (0.3–4 Hz), theta (4–8 Hz), lower alpha (8–12 Hz), higher alpha (12–15 Hz), lower beta (15–20 Hz), higher beta (20–30 Hz) frequency bands. The resulting filtered EEG data in each of the frequency bands is then stacked with the burst suppression timeline. This artificially creates a higher dimensional data set with more channels. The covariance matrices are estimated for each segment using the Oracle Approximating Shrinkage (OAS) method (Chen et al., 2010), ensuring that the covariance features are symmetric positive definite (SPD) matrices and therefore lie on the Riemannian manifold of SPD matrices (Supplementary Section S1.2). We identify further outlier segments by applying a variation of the Riemannian potato (Barachant et al., 2013; Barthélemy et al., 2019) on the covariances. The Riemannian potato method identifies outlier segments on the Riemannian manifold, by calculating the distance of the segment's covariance matrix on the manifold to a mean that adjusts over time. For covariance matrices that are too far away from the mean in the affine-invariant metric (Supplementary Section S1.2), we remove the corresponding segments from the spectral as well as the covariance data.

For both, the series of spectra and the covariance matrices, the corresponding segments are grouped by 2-min time frames, and then the mean is calculated for each time frame, giving a representation for every 2 min of the filtered operation.

### 2.2. Classification

After the preprocessing, we have two categories of data for classification, the EEG-based time series data, which are the covariance and spectral data, and the patient data with additional features extracted from the EEG recordings, namely the BSR and the LSP (Figure 1).

Since the data set exhibits a pronounced class imbalance (many more patients with than without POD), we use balanced ensemble methods, which can resolve this challenge (Hido et al., 2008; Galar et al., 2012). Hence, the patient data is classified by a balanced random forest classifier (Chen and Breiman, 2004). For the spectral and covariance data, in addition to the class imbalance, each patient has a different number of time frames depending on the length of their surgery. Therefore, we apply two sampling strategies for the two levels of imbalance. The sampling is embedded in a Bagging classifier, which uses support vector machines (SVMs) as a base estimator to classify individual time frames.

#### 2.2.1. Time-dependent features

There are two kinds of time-dependent features used in this study: the spectral and the covariance features. For each patient, there is a series of spectra and covariances calculated during preprocessing, which means we have a series of high-dimensional data as features for each patient. They represent a series of 2-min time frames from each patient's surgery. To deal with this data, we chose a Bagging approach, which deals with the different levels of data and imbalance: the patient level and the time frame level.

**Figure 2:**
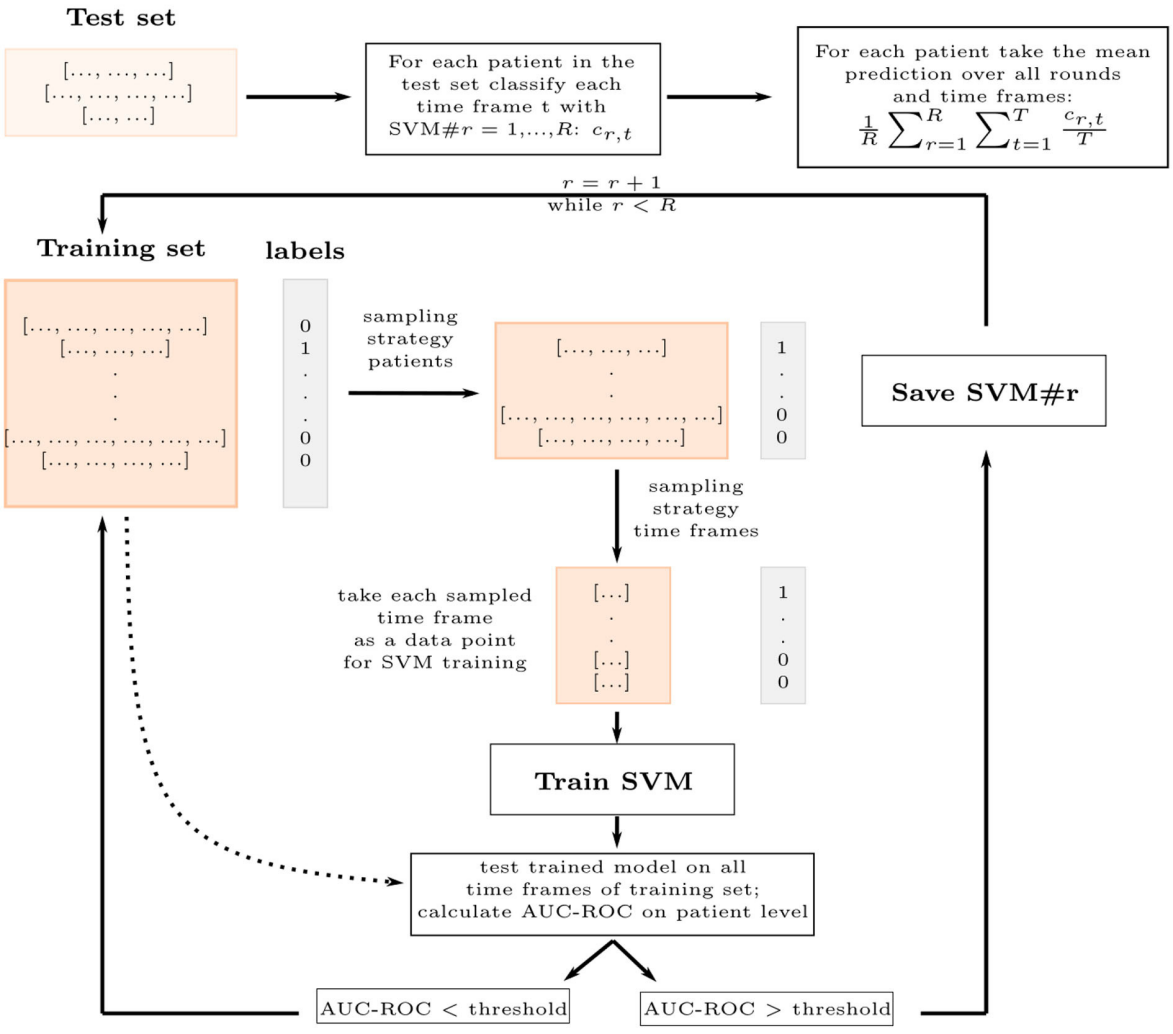
Bagging classifier schematic for time-dependent data. The Bagging approach saves *R* SVM estimators trained on the training set to classify all time frames in the test set and calculate a probability to develop POD for each patient in the test set. The SVMs are trained on sampled time frames as data points, the result of two stages of sampling on the training set: the strategy for patient sampling and the strategy for time frame sampling. The covariance features are projected to a Euclidean space before classification (Figure 3). If the AUC-ROC on the whole training set is good enough, the SVM is saved.

**Figure 3:**
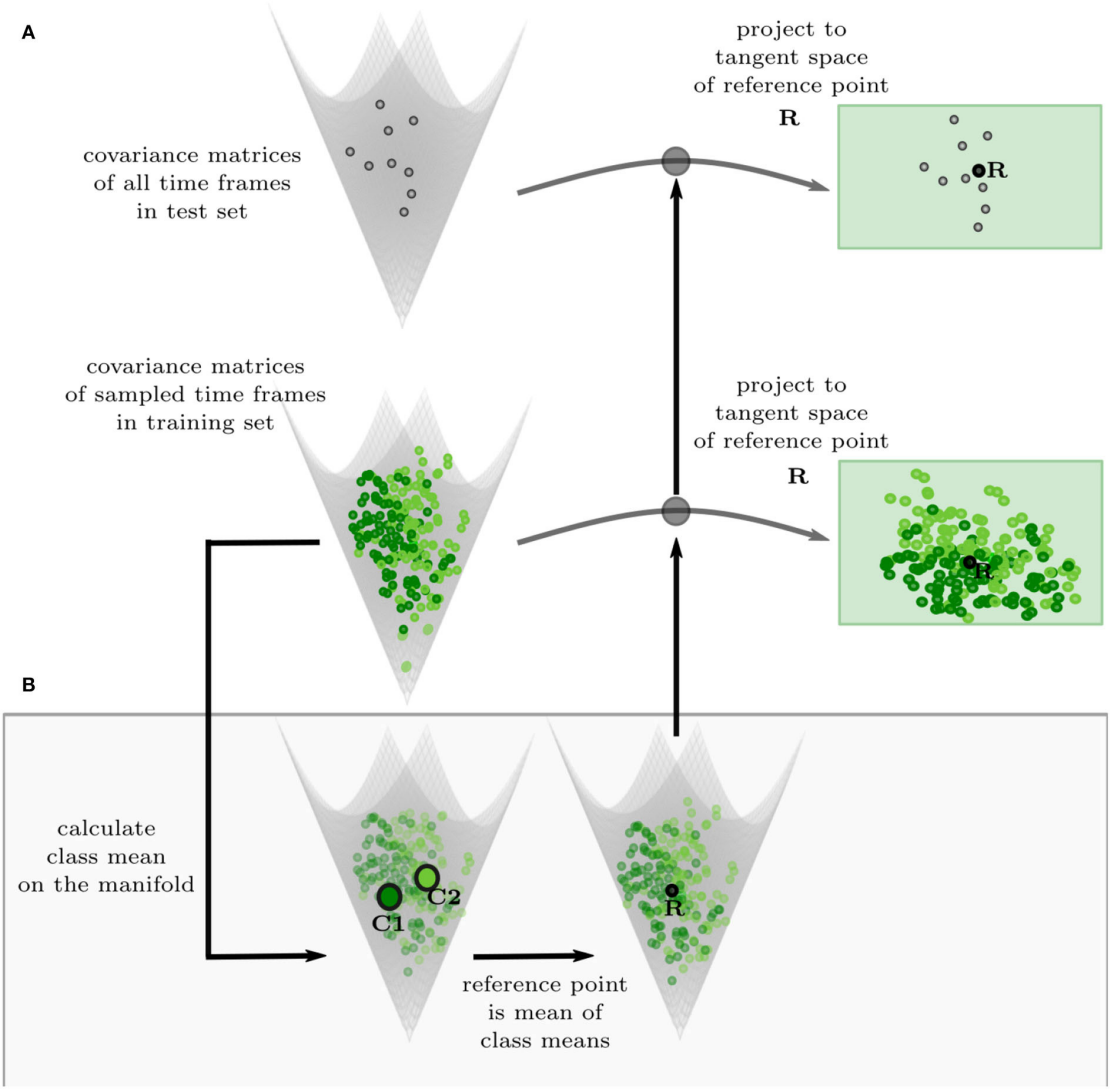
Projection of covariance features to the Euclidean tangent space. **(A)** The test set (gray, top) and the training set (green, bottom) are projected to the same tangent space of the reference point R. **(B)** The reference point R is calculated by taking the Riemannian mean of the Riemannian class means of the training set on the manifold, ensuring that the reference point is balanced regarding the different classes.

For training, we first undersample on the patient level, leaving out patients, then we undersample on the time frame level, leaving out time frames for the remaining patients (Figure 2). We, second, use each time frame as a separate data point for training a weighted SVM, keeping the patient label for each time frame corresponding to a patient. We train the SVM in the Euclidean space, which requires a few extra steps for the covariance matrices (Figure 3). Third, we check, whether a trained SVM reaches a set threshold on all time frames of the full data set and save the model to apply to the test set if it reached the threshold. Should the threshold not be reached within a set number of iterations, here 5, it is reduced by 2.5%. This is repeated until the number *R* of saved estimators is reached. The evaluation is done for all time frames *T*^*P*^ of the test patients *P*. The probability to develop POD is averaged over the time frames *T*^*P*^ and number of estimators *R* using the predicted classification $c_{r,t}^{P}$ for each time frame *t* from each estimator *r*. This results in the mean ratio of time frames that were classified as POD:


$$\begin{array}{l} p_{classifier}^{P}=\frac{1}{RT^{P}}\overset{R}{\underset{r=1}{\sum }}\overset{T^{P}}{\underset{t=1}{\sum }}c_{r,t}^{P} \end{array}$$

for both classifiers, the spectral classifier *spec* and the covariance classifier *cov*. We use this as the probability given by this classifier to predict whether a patient will develop delirium.

The sampling is done differently for the two types of time-dependent features. For both features, we randomly undersample the majority class on the patient level to reduce class imbalance, keeping at least $\frac{1}{3}$ of the majority class. For the spectral features, we then randomly sample a fixed number of time frames from each patient, ensuring, that patients with longer surgeries do not have a higher impact on the classification. For the covariance features, we sample again a fixed number of time frames, but the number depends on the class. We sample more time frames from the minority class, the POD patients, to make the data set more balanced and have a more diverse set of covariance matrices for the minority class. The latter, we found, improves classification, probably because a better reference point for the tangent space is found this way.

To be able to classify the covariance features with a SVM in the Euclidean space, they have to be projected into a euclidean space. Covariance matrices are SPD matrices, which form a Riemannian manifold, where a Riemannian metric describes the distance between two points (Supplementary Section S1.2). We can project the covariance features to a tangent space of the manifold, which is Euclidean, and there the SVMs are trained. The tangent space of a Riemannian manifold depends on the point, it is calculated from, the reference point, and is different at each point. Therefore, we find a reference point suitable for our training data and project the training and test set to the corresponding tangent space (Figure 3A; Congedo et al., 2017; Zanini et al., 2018). We chose a balanced Riemannian mean (Supplementary Section S1.2) of the sampled time frames as a reference point (Figure 3B). To calculate the balanced mean, the mean is first taken over the time frames of each class and then the mean of the two class means is calculated.

#### 2.2.2. Clinical and burst suppression features

The clinical features and the burst suppression features used in the patient and patient+burstsupp classifier respectively are numerical or categorical features for each patient. Therefore, this is a classical classification of imbalanced data per patient. We use a balanced random forest classifier (Chen and Breiman, 2004; Lemaître et al., 2017) for classification in both cases.

For patient data, we used only information that is readily available in the operating room. As such, we use which medications are used for anesthesia induction namely Propofol, Thiopental, or Etomidate, and which medications are used for the maintenance of anesthesia namely Propofol, Desflurane, Sevoflurane, or Isoflurane. Additionally, we use the ASA score, whether the patient received Benzodiazepine for premedication, the age of the patient, and the length of the operation. For the latter there is typically an estimation available before the operation, here we use the exact length. These are the features used in the patient data classifier.

The second classifier using the patient data to train adds the burst suppression signatures we extracted from the intraoperative EEG data to the patient data as additional features. This is the patient+burstsupp classifier. Specifically, we add the burst suppression ratio (BSR) and the longest suppression phase.

The full overview of the data used can be found in Table 1 (Supplementary Figures S1, S2). Significance was calculated by Pearson Chi-Square test for the ASA score and use of Benzodiazepines, and with the Kruskal-Wallis Test for age, surgery length, burst suppression ratio and mean longest suppression phase.

**Table 1:** Overview SuDoCo data.

	**No POD**				**POD**			
**Anesthetic agent for maintenance**	**Propofol**	**Desflurane (+Bolus)**	**Sevorflurane (+Bolus)**	**All**	**Propofol**	**Desflurane (+Bolus)**	**Sevorflurane (+Bolus)**	**All**
Number of patients	256	295	306	864	39	102	61	203
Age in years	68.9 ± 5.54	69.0 ± 6.23	69.6 ± 6.31	69.2 ± 6.06	71 ± 7.15	71.4 ± 6.65	73.4 ± 6.06	71.9 ± 6.61
ASA score	2.35 ± 0.57	2.44 ± 0.61	2.47 ± 0.586	2.41 ± 0.59	2.72 ± 0.56	2.56 ± 0.55	2.75 ± 0.54	2.65 ± 0.56
OP length in h	2.40 ± 1.39	2.86 ± 1.64	2.57 ± 1.52	2.61 ± 1.53	3.41 ± 1.79	4.31 ± 1.99	3.45 ± 1.97	3.88 ± 1.98
Benzo- diazepine	11	14	15	50	1	8	5	14
BSR	0.24 ± 0.08	0.182 ± 0.11	0.161 ± 0.09	0193 ± 0.1	0.260 ± 0.1	0.190 ± 0.12	0.169 ± 0.06	0.197 ± 0.1
Mean LSP in s	62.9	47.2	47.3	51.9	63.8	94.2	69.5	80.4

Overview of the data and groups used for classification. Desflurane and Sevoflurane include possible additional Propofol bolus. Values with significant *p*-values are marked in green. Significance was calculated by Pearson Chi-Square test for the ASA score and use of Benzodiazepines, and with Kruskal-Wallis Test for age, surgery length, burst suppression ratio, and mean longest suppression phase. The full table with all the *p*-values and additional data is available in Supplementary Table S1.

As is typical for random forest classifiers, the probability *p*_*classifier*_ for each patient *P* is based on the probability $p_{tree}^{P}$ given by each decision tree in the forest. Let *F* be the number of trees, then for the classifiers patient and patient+burst the probability for patient i to develop POD is given by $p_{classifier}^{P}=\frac{1}{F}\overset{F}{\underset{tree=1}{\sum }}p_{tree}^{P}$.

#### 2.2.3. Risk evaluation

To combine the three probabilities into a final risk evaluation, we calculate


(1) $$\begin{array}{l} p_{comb}=\max\left(p_{patient+burst},\min\left(p_{spec},p_{cov}\right)\right) \end{array}$$

for the probability prediction of the patient+burstsupp, spectral, and the covariance classifier. However, each of these classifiers can have a different optimal threshold for classification because the data is imbalanced and the classifiers are based on different averages taken. The optimal threshold for classification on the training set is the value, that maximizes the true positive rate (TPR) while minimizing the false positive rate (FPR) if every probability below the threshold is classified as negative and every probability above the threshold is classified as positive for POD (Calvert and Khoshgoftaar, 2019; Johnson and Khoshgoftaar, 2019; Zhang et al., 2020). This is solved by finding the threshold *th*_*classifier*_ that maximizes the g-mean $g=\sqrt{TPR\left(1-FPR\right)}$ for each classifier (Kubát and Matwin, 1997; Johnson and Khoshgoftaar, 2019). The risk evaluation then takes the optimal thresholds calculated on the training set into account. For each classifier, the optimal threshold *th*_*classifier*_ can be adjusted to $th_{classifier}^{shifted}=0.5$ by shifting the probabilities by *shift* = 0.5−*th*_*classifier*_, resulting in: $p_{classifier}^{shifted}=p_{classifier}+shift$. We test, how much the AUC-ROC score of *p*_*comb*_ improves on the training set when the probabilities are shifted to have an optimal threshold of 0.5 for all classifiers or two out of three. The shifts resulting in the highest AUC-ROC score of *p*_*comb*_ are saved and applied to the test set. If only two probabilities are shifted, the third shift is set to 0. Therefore, Equation (1) can be rewritten as $p_{comb}=\max\left(p_{patient+burst}^{shifted},\min\left(p_{spec}^{shifted},p_{cov}^{shifted}\right)\right).$

We chose to use the maximum formulation in the calculation for *p*_*comb*_ (1) to ensure a high negative predictive value (NPV). For the NPV calculated for the final results, the classification threshold is set at 0.5. To retain a better prediction for small values of *p*_*classifier*_, we set *p*_*comb*_ = *p*_*m*_, if *p*_*m*_ < 0.25, with *p*_*m*_ = *mean*(*p*_*classifier*_) being the common average of the probabilities for all classifiers (*patient* + *burst, spec, cov*) with a high enough AUC-ROC on the training set.

## 3. Results

From the initial 1,277 patients in the SuDoCo study, we excluded 210 patients, due to missing data (98) or EEG files shorter than 20 min after preprocessing (112). Patient characteristics of the included 1,067 patients are given in Table 1.

**Figure 4:**
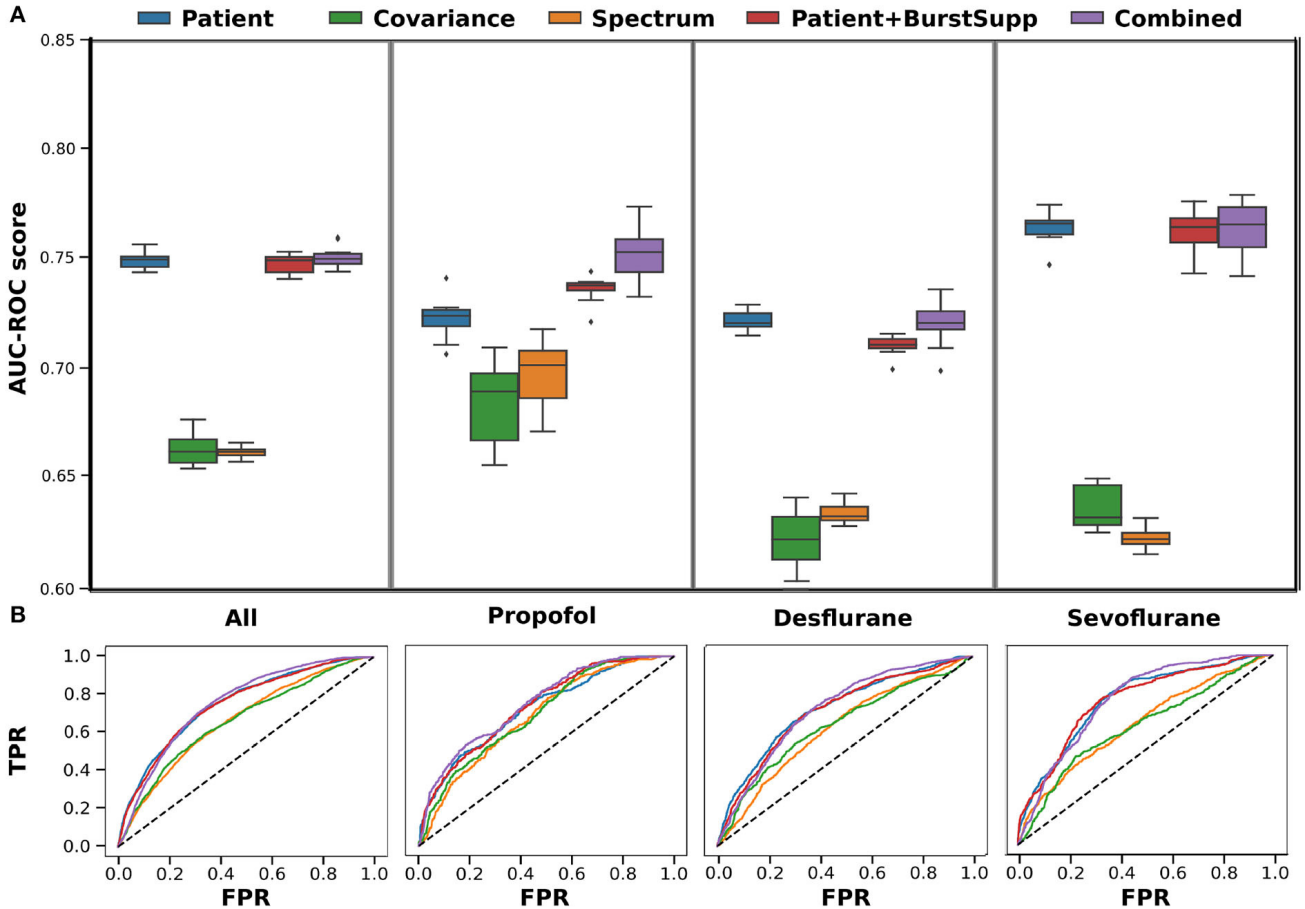
Cross-validation-results for training on all medications. Results shown for each medication group, left all, 2. Propofol, 3. Desflurane and right Sevoflurane, for each of the classifiers patient (features: anesthetic agent, benzodiazepine, ASA, Age, OP length) (blue), covariance (green), spectrum (orange), patient+burstsupp (patient and burst suppression features) (red), combined risk evaluation (violet). **(A)** ROC-AUC values in box plot over 10 repeats of 10-fold cross-validation. **(B)** Mean ROC-curves for 10 repeats of a 10-fold cross-validation.

When the data is trained on the whole data set, there is no apparent benefit of using EEG signatures for the classification. The results shown are the ROC curves and AUC-ROC scores over 10 repeats of a 10-fold cross-validation (Figure 4). The AUC-ROC score of the combined classifier is only 0.6% better than the patient classifier, and there is no improvement due to the burst suppression features. However, if we look at the different medications given for anesthesia maintenance, we see that the classifiers perform differently for the different medication groups. In particular, the EEG-based classifiers for the spectrum and the covariance perform significantly better for Propofol than for the inhalational anesthetics and the combined classifier improves over the patient classifier for Propofol.

**Figure 5:**
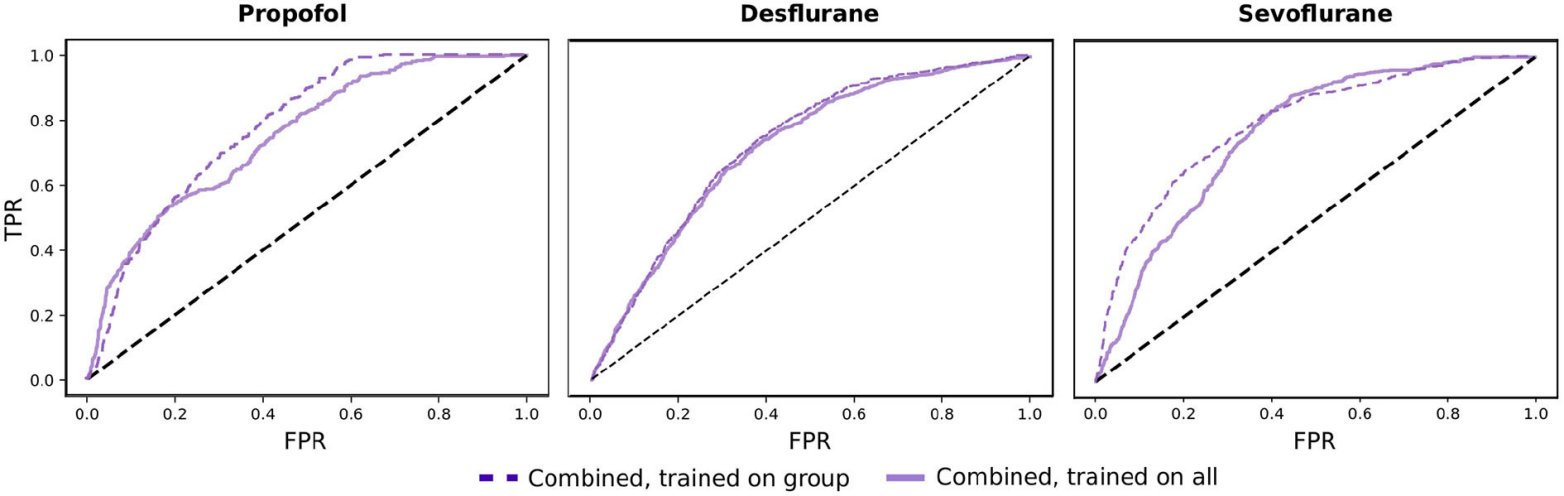
Comparison of combined risk evaluation. Results shown for Propofol, Desflurane, and Sevoflurane comparing the combined risk evaluation when the classifiers are trained on the corresponding medical group and when they are trained on the whole data set.

**Figure 6:**
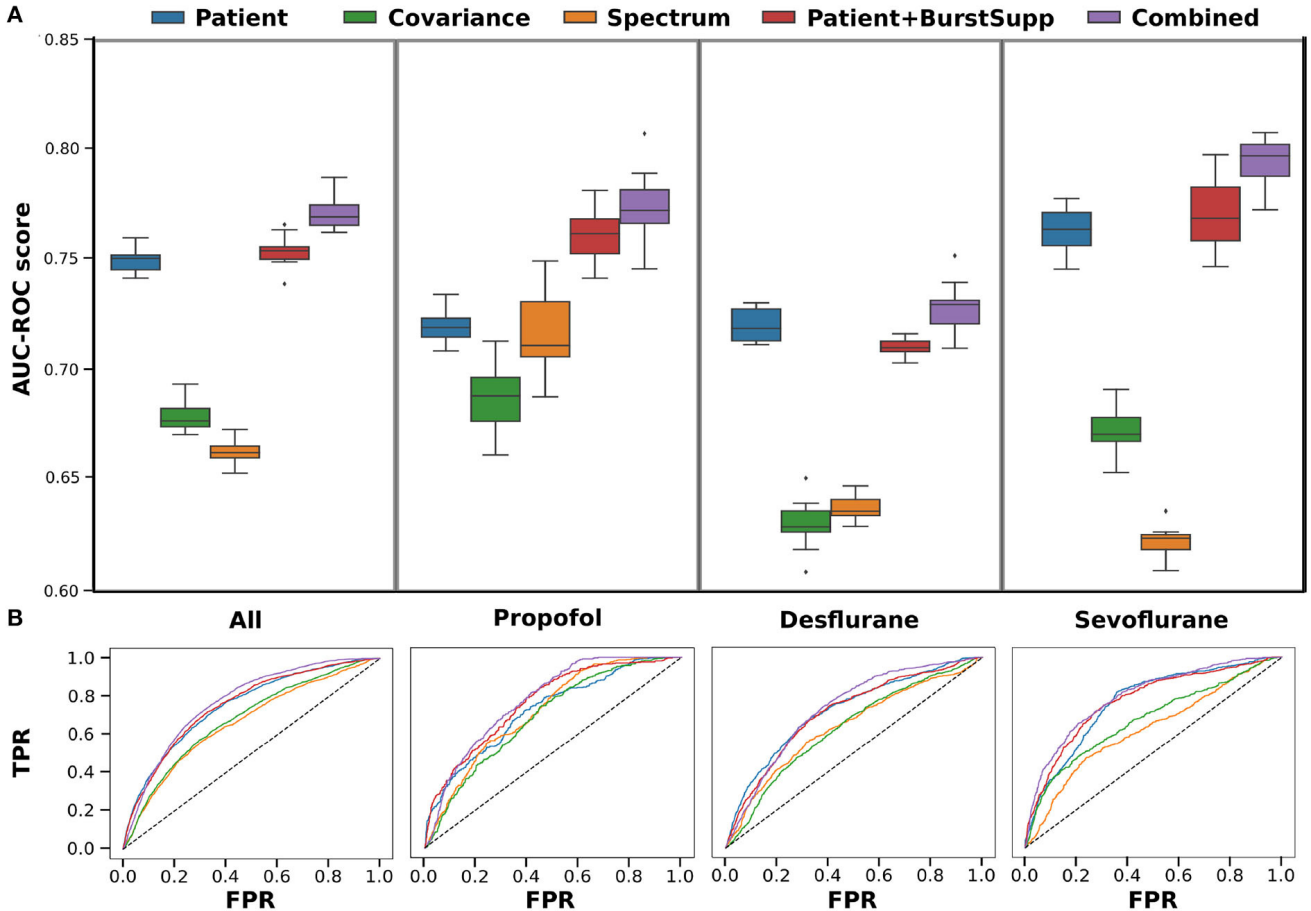
Cross-validation-results for training on group-specific data. Results shown for each medication group, left all, 2. Propofol, 3. Desflurane and right Sevoflurane, for each of the classifiers patient (features: anesthetic agent, benzodiazepine, ASA, Age, OP length) (blue), covariance (green), spectrum (orange), patient+burstsupp (patient and burst suppression features) (red), combined risk evaluation (violet). The medication group all includes all medications, the classifiers for each patient are based on the given medication group shown on the right. If patients do not fall into one of the groups, receiving other mixed medications, they are classified by the classifier trained on all medications. **(A)** ROC-AUC values in a box plot over 10 repeats of a 10-fold cross-validation. **(B)** Mean ROC-curves for 10 repeats of a 10-fold cross-validation.

Therefore, we investigated the medication groups and classifiers separately. For Propofol, the classification of the spectral features and burst suppression features improves. For Sevoflurane, the burst suppression features improve classification overall, while the covariance features improve by 4% (Supplementary Figure S3, **Figure 6**. Consequently, if the three groups are trained on separately, the performance of the combined risk evaluation improves for Sevoflurane and Propofol (Figure 5). For both medications, the addition of EEG signatures to the combined model improves the classification of POD.

Training the classifiers for the Propofol and Sevoflurane group on the corresponding training data, for which the classifiers perform best (Supplementary Figure S3), gives the best overall result (Figure 6). The spectral classifier for Propofol reaches an AUC-ROC score of 0.72 and the addition of burst suppression features to the patient data, improves the classification AUC-ROC by 0.05. For Sevoflurane the covariance classifier performs at 0.68 and the burst suppression features account for an improvement of 0.01 over the patient classifier. The combined risk evaluation is best in every medication group reaching a AUC-ROC of 0.78 for Propofol (NPV 0.92), 0.73 for Desflurane (NPV 0.89), 0.80 for Sevoflurane (NPV 0.92), and 0.77 (NPV 0.91) overall.

### 3.1. Validation on a separate study

To investigate the robustness of our classifiers, we used our models trained on the SuDoCo study data and classified the patients from a different study, the BioCog study. The BioCog study (BioCog project, 2014-2017) is a multicenter study, where intraoperative EEG recordings were done at the Charité-Universitätsmedizin Berlin (Campus Virchow Klinikum and Campus Mitte) from October 2014 until April 2017. Intraoperative raw EEG files were available from 78 patients. The EEG was recorded with the SEDline monitor (SEDline Root, Masimo, Irvine, USA) at the Fp1, Fp2, F7, and F8 electrode positions. We used the same preprocessing steps as included in our trained model, excluding six patients with missing data or with <20 min of EEG recording left after preprocessing (Table 2).

**Table 2:** Overview data BioCog.

	**No POD**				**POD**			
**Anesthetic agent for** **maintenance**	**Propofol**	**Desflurane (+Bolus)**	**Sevoflurane (+Bolus)**	**All**	**Propofol**	**Desflurane (+Bolus)**	**Sevoflurane (+Bolus)**	**All**
Number of patients	16	11	33	61	4	2	5	11
Age in years	71.9 ± 5.69	70.2 ± 3.46	71.8 ± 5.26	71.6 ± 5.04	71.3 ± 5.125	72.0 ± 1.41	76.6 ± 8.59	73.8 ± 6.69
ASA score	2.19 ± 0.54	2.09 ± 0.3	2.21 ± 0.42	2.18 ± 0.43	2.5 ± 0.58	2 ± 0.00	2.20 ± 0.84	2.27 ± 0.65
OP length in h	1.88 ± 1.57	2.61 ± 1.41	4.52 ± 3.5	2.54 ± 1.95	3.41 ± 1.79	4.31 ± 1.99	2.55 ± 1.45	4.04 ± 2.08
Benzo- diazepine	2	4	11	18	0	1	1	2
BSR								
	0.203 ± 0.16	0.151 ± 0.29	0.156 ± 0.22	0.158 ± 0.154	0.286 ± 0.16	0.266 ± 0.21	0.269 ± 0.45	0.268 ± 0.29
Mean LSP in s	150.1	59.6	62.3	86.5	597	15.1	162	293.3

Desflurane and Sevoflurane include possible additional Propofol bolus.

**Figure 7:**
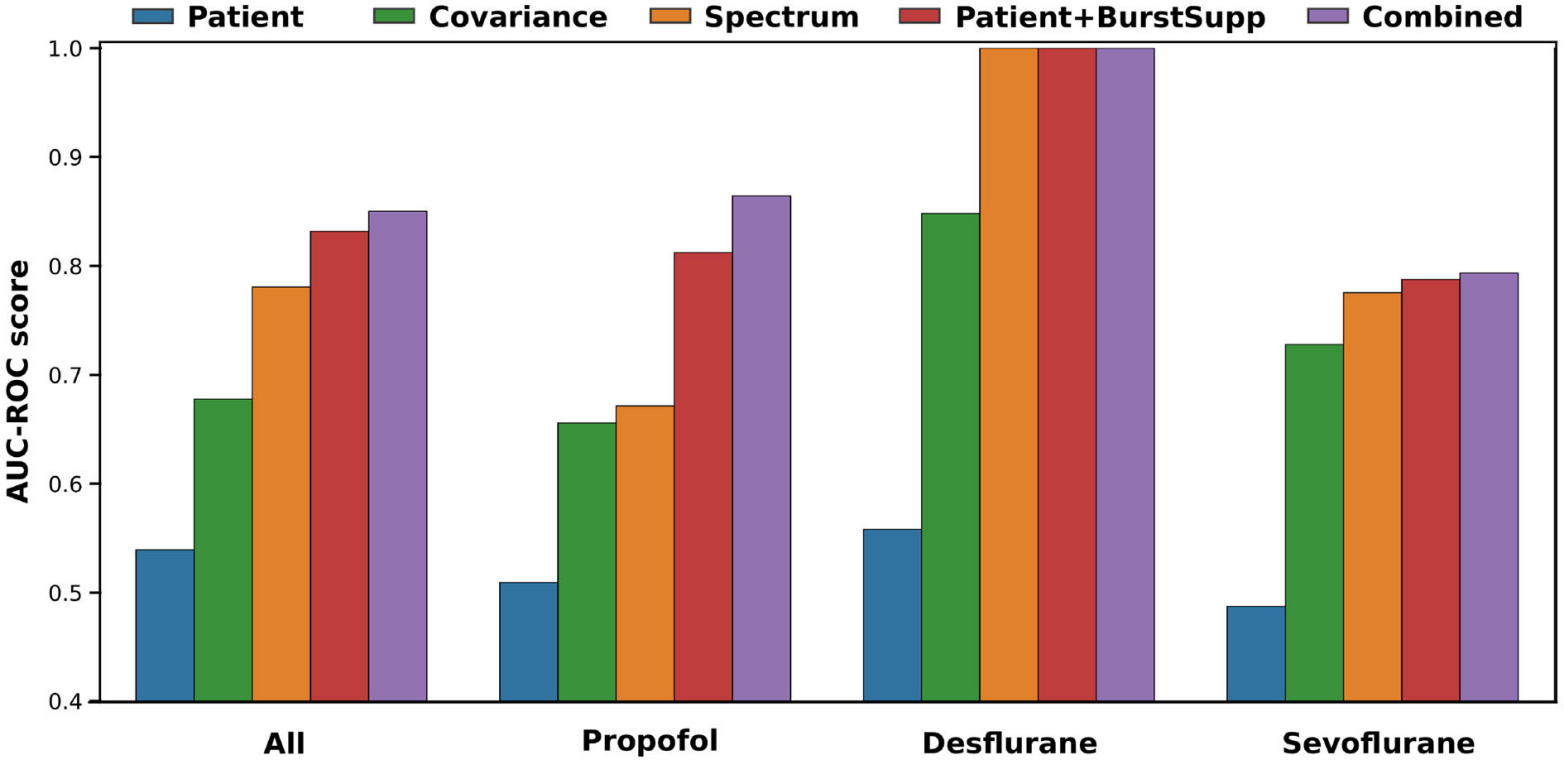
Validation results on BioCog study for a model trained on the group-specific data of the SuDoCo study. All (72 patients), Propofol (20 patients), Desflurane (13 patients), Sevoflurane (38 patients).

For each medication group, the patient+burstsupp and the combined classifier show a robust and good performance: an AUC-ROC score of 0.8 for Sevoflurane, 0.87 for Propofol, and 1.0 for Desflurane, 0.85 for all medications (Figure 7). The patient data classifier does not show these results. The EEG-based classifiers alone, however, perform fairly well. To use the covariance classifier, we project the new data covariance matrices on the manifold to the saved reference point (Zanini et al., 2018, Supplementary Section S3). We correct the probability results for each group and classifier to an optimal threshold of 0.5 with the optimal thresholds calculated on the SuDoCo training set (Section 2.2.3).

## 4. Discussion

Our results indicate that including routine EEG measurements in a POD risk evaluation gives a robust and good prediction both in cross-validation and in a separate study. Both studies include older patients without any focus on a specific surgical population. The combined classifier, incorporating patient data and signatures from the intraoperative EEG monitoring, outperforms any of the approaches using only one of the two.

Using only our limited patient data appears to be sensitive to small shifts in the patient data, as we can see for our validation study, where the Age is generally older while the ASA score is lower than in the SuDoCo study (Tables 1, 2, Supplementary Figures S2, S3). Including the EEG signatures made the results more robust to shifts in the patient data, even though the EEG was recorded with a different monitor.

Adding markers to the EEG for the beginning and end of the operation, specifically at the first cut and the last stitch, might also improve the results by making the preprocessing more precise. Generally, we cannot make any claims about other combinations of anesthetic agents because there were very few instances in the training set. Furthermore, while the transfer of the model to the validation study shows proof of concept and robustness, the very good results on the validation set, especially for Desflurane, might be the result of the few such patients in the validation set. The 38 Sevoflurane patients in the validation set, however, show results closer to the cross-validation results, which was expected (Table 2).

We show that taking the medication into account can improve the results of the overall risk evaluation, by improving the classifiers using the EEG features. This is probably partly due to the different class imbalances, for the different medication groups (Tables 1, 2). The main reason, though, is the effect on the EEG of the different medications. Taking away those differences should increase the similarities of the training and test set. However, contrary to our expectations, this did not work for every medication group. The effect might be counteracted by the fact, that we make the training set smaller when we look at the groups separately, which explains why the approach only worked partly. From literature (Soehle et al., 2015), one would expect the burst suppression features to improve the prediction for all medication groups, which did not work for the Desflurane group in cross-validation. However, it did improve the transfer to a new dataset for all medications. The study did not include the dosage of the anesthetic agent given, this might have weakened our results since the sensitivity to anesthetic agents varies within the older population (Cooter Wright et al., 2022).

Even when including little prior knowledge about the patient, the cross-validation results for Propofol and Sevoflurane are comparable to the POD prediction results achieved by Bishara et al. (2022), using electronic health record data of 24,885 adults, reporting an average AUC-ROC of 0.82 for older patients. This shows the potential of incorporating EEG monitoring data into machine learning algorithms to predict POD. Unfortunately, there are very few publications for older patients of a general surgical population. A Random Forest classifier in Wang et al. (2020) reached an AUC-ROC score of 0.96 for 912 patients undergoing microvascular decompression surgery, trained on patient data features, and validated on a test set. A logistic regression model trained on MRI data (Kyeong et al., 2018) predicted POD for 57 older patients with a femoral neck fracture with a cross-validation AUC-ROC of 0.92. However, collecting the required data before surgery is time-consuming and expensive. Limiting the patient data to readily available information, without any additional examinations or measurements, is more practical.

Generally, one has to be mindful of biases in the prediction. While the risk evaluation allows focusing efforts on a group with high risk in a busy clinical ward, it disadvantages false negative patients. This is why our risk evaluation favors false positives. The limited clinical data combined with the EEG data, we believe, are less prone to societal biases than other clinical parameters. Nevertheless, the training set is imbalanced and before introducing any such approach into a clinical setting, one would need to investigate carefully, if there are any relevant biases and how to address them.

Our approach remains easily applicable because the patient data used is available in the operating room. EEG is already used routinely to monitor the depth of anesthesia and therefore widely available and affordable. Incorporating the EEG signatures into a POD prediction method has the potential to improve the overall results and robustness.

## 5. Conclusion

Incorporating EEG data into a machine learning approach gives a reliable risk evaluation for POD. We could show that adding EEG signatures to the approach improves classification compared to using limited patient data with classical risk factors, such as age, the ASA score, and the length of the operation. However, this only works, if we take the different maintenance medications into account because they affect the EEG. Including the medication in EEG monitors might, therefore, generally be useful. For future work, it might be interesting to investigate Desflurane a little closer, since the POD incidence in that group is higher than in the other groups and burst suppression signatures do not seem to be a relevant risk factor for most of these patients. The patient information we use is limited to six features and could quickly be typed into future monitoring equipment. Our approach shows that using EEG data in a machine learning approach could be used as a software tool in EEG monitors to give a risk evaluation for POD at the end of surgery.

## Data availability statement

The data analyzed in this study is subject to the following licenses/restrictions: Due to the nature of this research, participants of the original study did not agree for their data to be shared publicly, so supporting data is not available. The code is available in the Supplementary material. Requests to access these datasets should be directed to SK, susanne.koch@charite.de.

## Author contributions

BB, CS, and SK conceived of the presented idea. FR coordinated the original study. VR derived the methods and performed the computations and analysis. VR, BB, and SK discussed the results and contributed to the final manuscript in consultation with FR and CS. All authors contributed to the article and approved the submitted version.

## Funding

VR acknowledges the financial support from the Research Training Group (RTG 2433) DAEDALUS (Differential Equation- and Data-driven Models in Life Sciences and Fluid Dynamics) funded by Deutsche Forschungsgemeinschaft (DFG, German Research Foundation, project 384950143). We acknowledge support from the German Research Foundation and the Open Access Publication Fund of TU Berlin.

## Conflict of interest

Author SK is an inventor on patents, sold to Medtronic. She reports a grant during the conduct of the study by the German Research Foundation. Author CS is an inventor on patents, she reports grants during the conduct of a study from the European Commission, from Aridis Pharmaceutical Inc., B. Braun Melsung, Drägerwerk AG & Co. KGaA, German Research Foundation, German Aerospace Center, Einstein Foundation Berlin, European Society of Anaesthesiology, Federal Joint Committee and Inner University grants. Grants promoting Science and Education from WHOCC, Baxter Deutschland GmbH, Cytosorbents Europe GmbH, Edwars Lifesciences Germany GmbH, Fresenius Medical Care, Grünenthal GmbH, Masimo Europe Ltd. Phizer Pharma PFE GmbH. Personal fees from Georg Thieme Verlag, Dr. F. Köhler Chemie GmbH, Sintetica GmbH, European Commission, Stifterverband für die deutsche Wissenschaft e.V./Philips, Stiftung Charite, AGUETTANT Deutschland GmbH, AbbVie Deutschland GmbH & Co. KG, Amomed Pharma GmbH, Touch Health, Copra System GmbH, Correvio GmbH, Max-Planck-Gesellschaft zur Förderung der Wissenschaft e.V., Deutsche Gesellschaft für Anästhesiologie & Intensivmedizin (DGAI), Medtronic, Philips Electronics Nederland BV, BMG, and BMBF. Aspect Medical Systems, now Medtronic, funded the initial SuDoCo study were CS was PI. The funders had no role in study design, data collection and analysis, decision to publish, or preparation of the manuscript. The remaining authors declare that the research was conducted in the absence of any commercial or financial relationships that could be construed as a potential conflict of interest.

## Publisher's note

All claims expressed in this article are solely those of the authors and do not necessarily represent those of their affiliated organizations, or those of the publisher, the editors and the reviewers. Any product that may be evaluated in this article, or claim that may be made by its manufacturer, is not guaranteed or endorsed by the publisher.
