# Supplementary material for: Machine-learning model predicting postoperative delirium in older patients using intraoperative frontal electroencephalographic signatures
Source: Front Aging Neurosci. 2022 Oct 14;14:911088. doi: 10.3389/fnagi.2022.911088 (PMC9614270; doi:10.3389/fnagi.2022.911088)
Supplement: Supplementary file 1 [file Data_Sheet_1.PDF]

Supplementary Material

S1 METHODS

All the functions and parameters used, can be found under `https://github.com/roehrver/PODprediction`.

S1.1 Artifact Removal and Automatic Segmentation

Due to the nature of our EEG data, some of the classical EEG artifacts, such as blinking, are not as prominent as others. The patients have their eyes closed and they receive a muscle relaxant, which reduces muscle and blinking artifacts. However, technical and environmental artifacts, e.g. caused by regular blood pressure measurements or the patient being moved remain. Rapid eye movement is also likely being recorded, especially because the monitoring only has frontal EEG channels. Due to the limited number of channels, we do not use spatial preprocessing methods.

We instead focus on two main types of artifacts, high amplitude artifacts, and frequency artifacts. For the first category, we use an amplitude filter, removing high amplitude artifacts by choosing the 99% quantile of the amplitudes as a threshold. For each time point that is excluded, we add a buffer interval of 1 s around the time point and set the signal at that point to 0. (Fig. S1) We then re-reference the data by the common average reference. For the second category we apply a Bandpass filter (Butterworth filter, 2. order, Butterworth (1930)), filtering out the signal above 50 Hz, to reduce electrical line noise, and below 0.3 Hz, to combat drifts. To make sure the line noise is filtered out more thoroughly, one could set the cut-off frequency to 47 Hz for future research or use an additional notch filter. This did not impact the classification accuracy in this study.

Band-pass filters introduce filtering artifacts on jumps in the data, the edge artifacts. They can be easily spotted in the zero line, where the high amplitude artifacts were removed. (Fig. S1, Bandpass filter) The edge artifacts were removed by deleting the marked time points and the buffer interval from the data, leaving us with a series of separated time segments. This ensures, that we removed any edge artifacts introduced by the filter as well as any potential residual artifacts connected temporally to the high amplitude artifacts. Any segments smaller than 1 s are removed from the series, to ensure an accurate estimation of the spectrum and covariance matrix for each segment.

We identify further outlier segments by applying a variation of the Riemannian potato (Barthélemy et al. (2019); Barachant et al. (2013)) on the covariances and remove the corresponding segments from the spectral as well as the covariance data. The Riemannian potato excludes segments, that do not fulfill the following condition: The covariance matrix is below a threshold far away from a mean based on the Riemannian distance on the manifold. (Sec. S1.2) In the beginning, the mean is set to the mean of the covariances of the first $X = 180$ segments. The mean is moving: for each segment satisfying the condition, it is moving a fraction of the way along the geodesic towards that segment's covariance matrix. The threshold is defined by the 97.5% quantile of Riemannian distances of the X segments surrounding the current one.

S1.2 Riemannian Geometry of SPD matrices

Symmetric positive definite (SPD) matrices $\mathbb{P}_n$, such as the covariance matrices we work with, have a smooth Riemannian manifold structure. It is a convex cone without a boundary in the $\mathbb{R}^{n\times n}$. The structure

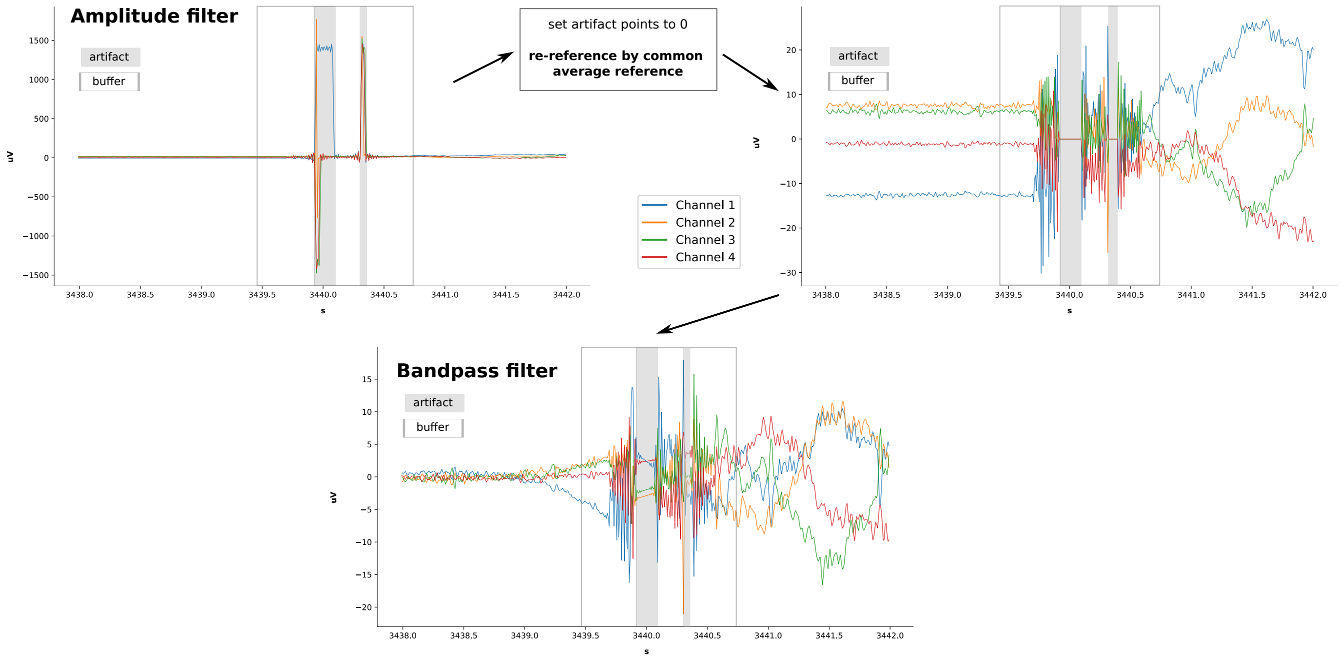

Figure S1. Artifact filtering. This shows the first preprocessing steps on an example time interval for all four channels of one patient. 1. The amplitude filter finds high amplitude artifacts and marks them as well as a buffer of one second around them. 2. the amplitude artifacts are set to 0 and the data is re-referenced. 3. A bandpass filter is applied to remove frequency artifacts. Any edge artifacts, introduced by the filter lie well within the buffer and are removed after this step.

is defined by a scalar product, its metric, and the corresponding geodesic. It allows not only for a definition of a distance and therefore e. g. for the possibility to average on the manifold, but also to define a tangent space and differentials. Introducing all the concepts of Riemannian geometry for SPD matrices would exceed the scope of this paper, so we will stick to the basic concepts directly related to what we used in this study. A good reference is ”Positive Definite Matrices” (Bhatia (2007)).

We use the affine-invariant or Fisher-Rao metric $\left\| log(P^{1/2}QP^{1/2}) \right\|$, which defines a metric on the manifold. (Congedo et al. (2017)) The corresponding geodesic is $\gamma_{P,Q}(t) = P^{-0.5}(P^{-0.5}QP^{-0.5})^t P^{-0.5}$ and defines the shortest path or curve on the manifold between two points P,$Q \in \mathbb{P}_n$. For $t = 0.5$ for example, one has found the halfway point or mean between the two points. To find the Riemannian mean M for $[P_1, ..., P_k]$ one has to solve the equation $0 = \sum_{i=1}^{k} \log(M^{-0.5} P_i M^{-0.5})$, which is done through a gradient descent algorithm (Congedo et al. (2015)).

The scalar product corresponding to this Riemannian metric is $g_P^R(A, B) = tr(P^{-1}AP^{-1}B)$ with $P \in \mathbb{P}_n$ and A,$B \in T_P\mathbb{P}_n$, the tangent space of $\mathbb{P}_n$ in the point P on the manifold. The tangent space depends on the reference point P. To map a point Q from the manifold to the tangent space, we can use the logarithmic map $Log_P(Q) = P^{0.5} \log(P^{-0.5}QP^{-0.5})P^{0.5}$. Hence, the matrices on the tangent space are symmetric.

S1.3 Patient+Burst Suppression Features

Calculating the burst suppression features relies on a two-step process. First, we define a threshold $T1$, dependent on the EEG data distribution, to sort the data into two categories of amplitudes, represented

by 1 (more likely suppression) and 0 (more likely not suppression). $T1$ is set at the 60% quantile of the absolute values of amplitudes for this study. Then we take a one-second window around each time point and calculate the mean over the binary signal in that window, this is burst suppression probability at that point, creating the burst suppression timeline overall points. The window is used to account for burst suppression being a pattern over sustained amounts of time. Since we only excluded segments that were smaller than 1 s, we set the range to 1 s. A longer period would be reasonable, if possible. Any values higher than a second threshold $T2$ $(= 0.8)$, i.e., when at least 80% of the sample points within the time window have a low amplitude, will be categorized as *suppression time points*.

The suppression duration in s is the number of all the suppression time points divided by the sample rate. The burst suppression ratio is the ratio of the suppression duration and the length of the artifact-reduced operation in seconds. The longest suppression phase finds the longest consecutive series of suppression time points.

Figures S2 and S3 show that the the features do not clearly separate. The general age in the BioCog study is higher, while the ASA score is generally lower than in the SuDoCo study. One can also see a few outliers in the burst suppression features, which is probably due to the thresholding not being optimal for those patients. For future research, one could dynamically adjust the threshold not only to the EEG data distribution but change the threshold quantile, if unrealistic values occur and potentially adjust for the medication already.

S2 COMPARISON RESULTS TRAINED ON WHOLE SUDOCO DATA SET VS ON THE GROUPS

We chose which group to train the model on, according to the following comparison (Fig. S4), done for the different classifiers on the different medication groups. We chose to train the Desflurane group on the whole data set, the Sevoflurane group on the whole data set for the spectral classifier, and on the group for the covariance and the patient+burstsupp classifier. And finally, the Propofol group is trained on the group for the spectral and patient+burstsupp classifier and on the whole data set for the covariance classifier.

S3 SHIFT OF COVARIANCES IN BIOCOG DATA SET

Since the BioCog study uses a different monitor for the EEG data, we have to project the BioCog data covariance matrices on the manifold to the saved anchor point in order to then project the data onto the previously calculated tangent space (Zanini et al. (2018)). We do not have two classes per patient, which is the norm for classical brain-computer-interface tasks, hence we cannot project each patient relative to their own mean to the anchor point. This would lead to the data points of both classes and all patients overlapping because they were projected to the same point on the manifold. Therefore, we need an external reference for the new data set. Since there are comparably few patients, we use the other patients as references for each patient. We calculate the balanced geometric mean for those patients and take this as the reference point, which is projected directly onto the anchor point of the tangent space. The data of the patient that is being classified is then projected relative to this reference point to the neighborhood of the anchor point. This is done for each patient in the BioCog data set.

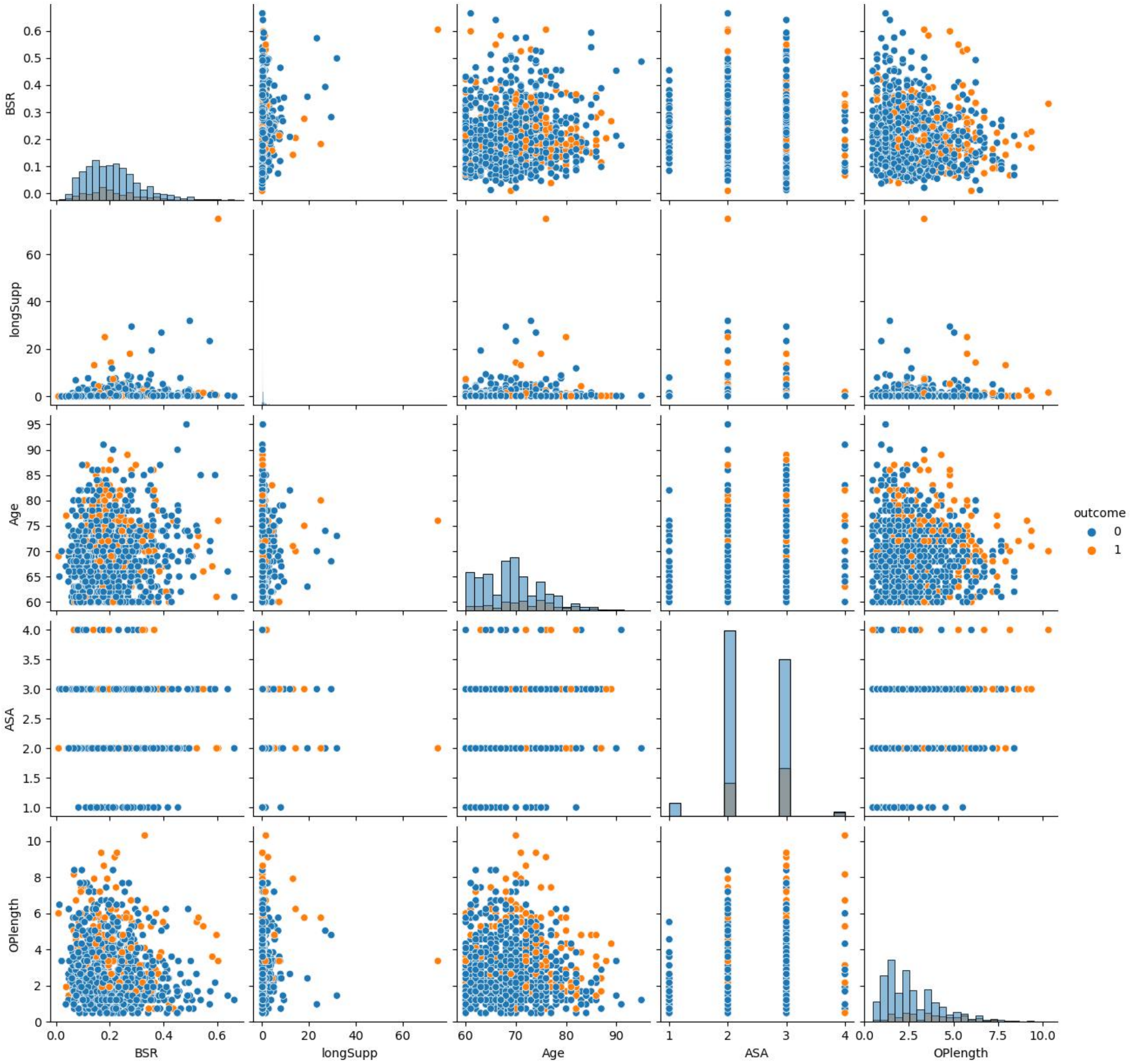

Figure S2. Relations and distributions of the patient and burst suppression feature of the SuDoCo study. The following features are displayed for all patients with respect to the outcome (0/blue: No POD, 1/orange: POD): BSR, longest suppression phase (in min), Age (in years), ASA score, operation length (in hours). The order from left to right and top to bottom is the same. The subplots on the diagonal show the distribution of each feature. The other subplots show the relations between the different features in scatter plots, each dot representing one patient. (The plot was generated using the Seaborn pairplot function.Waskom (2021); Hunter (2007))

REFERENCES

Butterworth S. On the theory of filter amplifiers. *xperimental Wireless and the Wireless Engineer* **7** (1930) 536–541.

Barthélemy Q, Mayaud L, Ojeda D, Congedo M. The riemannian potato field: A tool for online signal quality index of eeg. *IEEE Transactions on Neural Systems and Rehabilitation Engineering* **27** (2019)

	No POD				POD			
Anesthetic agent for maintenance	Propofol	Desflurane (+Bolus)	Sevorflurane (+Bolus)	All	Propofol	Desflurane (+Bolus)	Sevorflurane (+Bolus)	All
number of patients	256	295	306	864	39	102	61	203
sex (m/f)	35%/ 65% *(0.469)*	53%/ 47% *(0.137)*	69.5%/ 30.5% *(0.031)*	54%/ 46% *(0.962)*	29%/ 71% *(0.469)*	62%/ 38% *(0.137)*	55%/ 45% *(0.031)*	53.5%/ 46.5% *(0.962)*
Age in years	68.9 ±5.54 *(0.039)*	69.0 ±6.23 *(0.001)*	69.6 ±6.31 *(0.000)*	69.2 ±6.06 *(0.000)*	71 ±7.15 *(0.039)*	71.4 ±6.65 *(0.001)*	73.4 ±6.06 *(0.000)*	71.9 ±6.61 *(0.000)*
ASA score	2.35 ±0.57 *(0.004)*	2.44 ±0.61 *(0.163)*	2.47 ±0.586 *(0.009)*	2.41 ±0.59 *(0.000)*	2.72 ±0.56 *(0.004)*	2.56 ±0.55 *(0.163)*	2.75 ±0.54 *(0.009)*	2.65 ±0.56 *(0.000)*
OP length in h	2.40 ±1.39 *(0.002)*	2.86 ±1.64 *(0.000)*	2.57 ±1.52 *(0.002)*	2.61 ±1.53 *(0.000)*	3.41 ±1.79 *(0.002)*	4.31 ±1.99 *(0.000)*	3.45 ±1.97 *(0.002)*	3.88 ±1.98 *(0.000)*
Benzo-diazepine	11 *(0.688)*	14 *(0.243)*	15 *(0.303)*	50 *(0.163)*	1 *(0.688)*	8 *(0.243)*	5 *(0.303)*	14 *(0.163)*
BSR	0.24 ±0.08 *(0.440)*	0.182 ±0.11 *(0.776)*	0.161 ±0.09 *(0.432)*	0193 ±0.1 *(0.839)*	0.260 ±0.1 *(0.440)*	0.190 ±0.12 *(0.776)*	0.169 ±0.06 *(0.432)*	0.197 ±0.1 *(0.839)*
mean longest supp. phase in s	62.9 *(0.958)*	47.2 *(0.411)*	47.3 *(0.364)*	51.9 *(0.827)*	63.8 *(0.958)*	94.2 *(0.411)*	69.5 *(0.364)*	80.4 *(0.827)*

Table S1. Overview SuDoCo Data Including the sex of patients for completeness. Desflurane and Sevoflurane include possible additional Propofol Bolus. p-values are given in (), significant p-values (< 0.05) are marked in green. The p-values were calculated by Pearson Chi-Square test for sex, ASA score, and use of Benzodiazepines, and with Kruskal-Wallis Test for age, surgery length, burst suppression ratio and mean longest suppression phase.

244–255. doi:10.1109/TNSRE.2019.2893113.

Barachant A, Andreev A, Congedo M. The riemannian potato: an automatic and adaptive artifact detection method for online experiments using riemannian geometry. *TOBI Workshop lV* (2013).

Bhatia R. *Positive Definite Matrices* (Princeton University press.) (2007).

Congedo M, Barachant A, Bhatia R. Riemannian geometry for eeg-based brain-computer interfaces; a primer and a review. *Brain-Computer Interfaces* **4** (2017) 1–20. doi:10.1080/2326263X.2017.1297192.

Congedo M, Afsari B, Barachant A, Moakher M. Approximate joint diagonalization and geometric mean of symmetric positive definite matrices. *PLOS ONE* **10** (2015) 1–25. doi:10.1371/journal.pone.0121423.

Waskom ML. seaborn: statistical data visualization. *Journal of Open Source Software* **6** (2021) 3021. doi:10.21105/joss.03021.

Hunter JD. Matplotlib: A 2d graphics environment. *Computing in Science & Engineering* **9** (2007) 90–95. doi:10.1109/MCSE.2007.55.

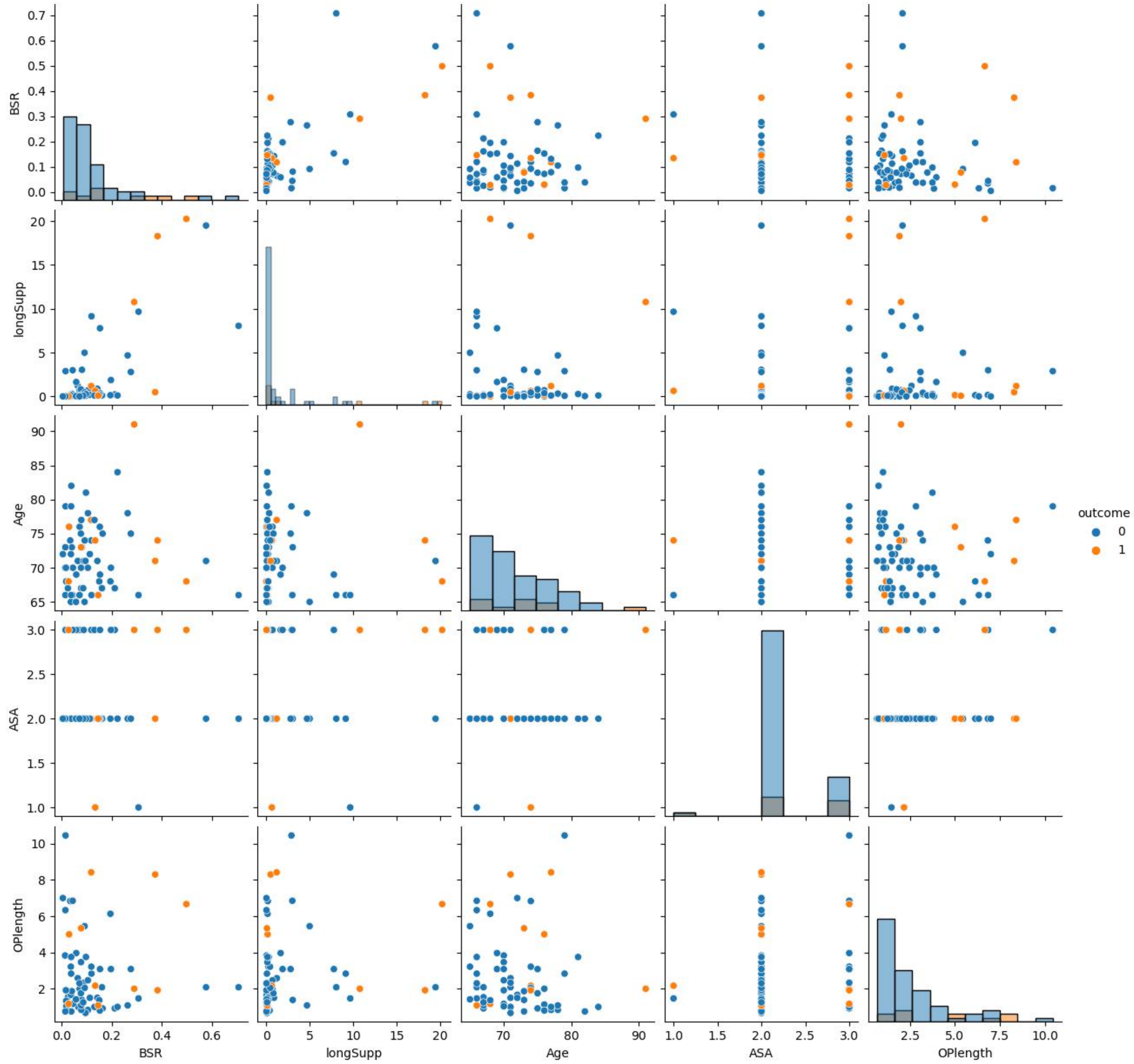

Figure S3. Relations and distributions of the patient and burst suppression feature of the BioCog study. The following features are displayed for all patients with respect to the outcome (0/blue: No POD, 1/orange: POD): BSR, longest suppression phase (in min), Age (in years), ASA score, operation length (in hours). The order from left to right and top to bottom is the same. The subplots on the diagonal show the distribution of each feature. The other subplots show the relations between the different features in scatter plots, each dot representing one patient. (The plot was generated using the Seaborn pairplot function.Waskom (2021); Hunter (2007))

Zanini P, Congedo M, Jutten C, Said S, Berthoumieu Y. Transfer learning: A riemannian geometry framework with applications to brain–computer interfaces. *IEEE Transactions on Biomedical Engineering* **65** (2018) 1107–1116. doi:10.1109/TBME.2017.2742541.

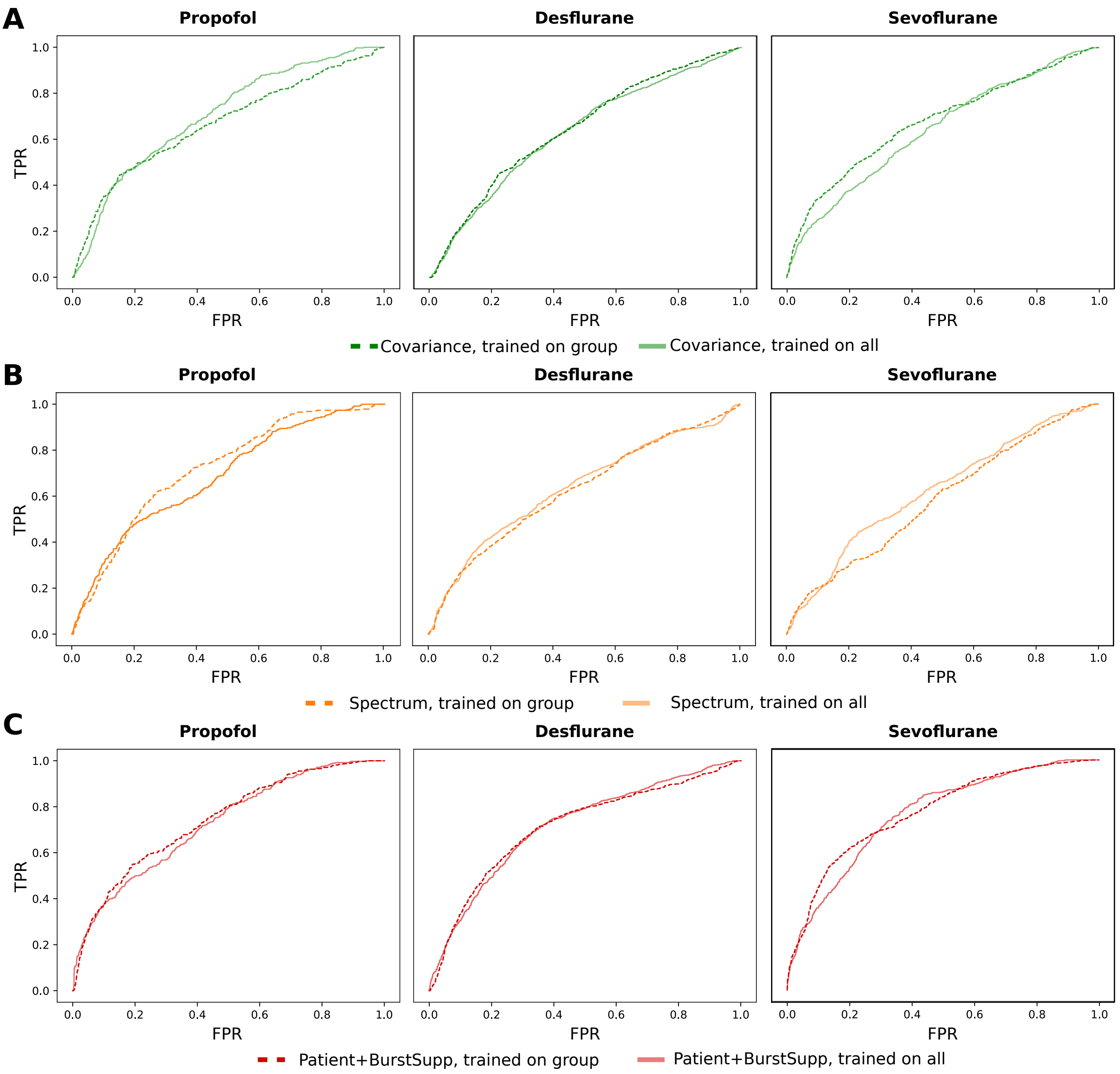

Figure S4. Comparison Results trained on whole SuDoKo Data Set vs on the medication groups: (A) covariance classifier, (B) spectral classifier, (C) patient+burstsupp
